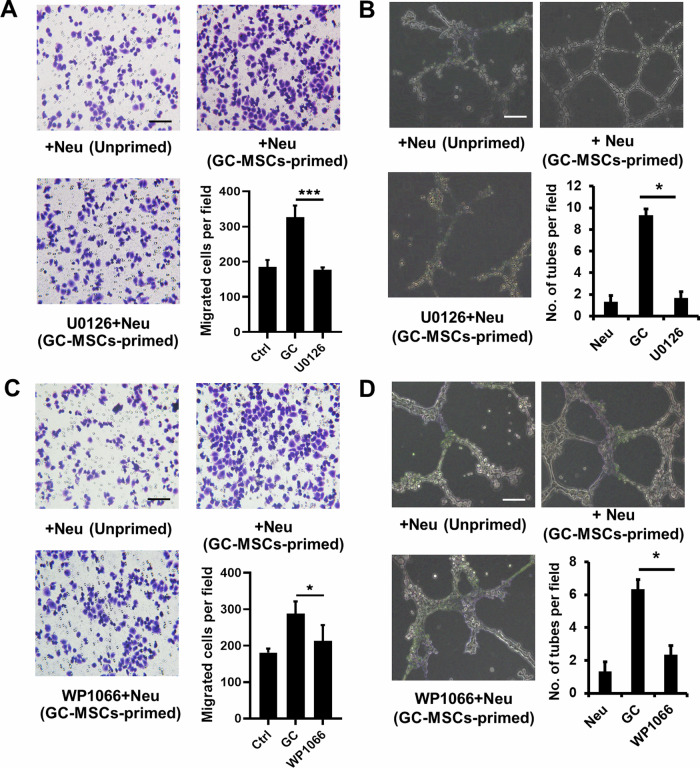# Correction to: The IL-6-STAT3 axis mediates a reciprocal crosstalk between cancer-derived mesenchymal stem cells and neutrophils to synergistically prompt gastric cancer progression

**DOI:** 10.1038/s41419-024-07100-y

**Published:** 2024-10-01

**Authors:** Q. Zhu, X. Zhang, L. Zhang, W. Li, H. Wu, X. Yuan, F. Mao, M. Wang, W. Zhu, H. Qian, W. Xu

**Affiliations:** 1 School of Medical Science and Laboratory Medicine, Jiangsu University, Zhenjiang, Jiangsu China; 2 Department of Central Laboratory, The First People’s Hospital of Lianyungang, Lianyungang, Jiangsu China; 3 The Affiliated Hospital, Jiangsu University, Zhenjiang, Jiangsu China

Correction to: *Cell Death and Disease* 10.1038/cddis.2014.263, published online 19 June 2014

The authors regret that a mistake was found in Figure 4. The top right panels of Figure 4a and Figure 4c had partial overlapping as they came from the same group. This mistake was due to our carelessness in using the images. We feel very sorry for our carelessness, and apply to correct the Figure 4.

The authors would like to apologize for any inconvenience caused.

Original figure 4
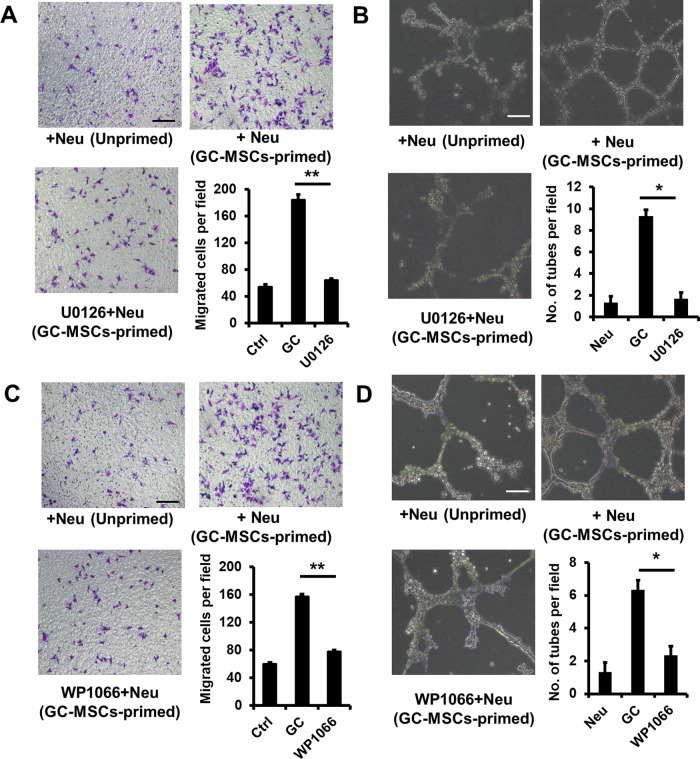


Corrected figure 4